# Effect of basal metabolic rate on osteoporosis: A Mendelian randomization study

**DOI:** 10.3389/fpubh.2023.1096519

**Published:** 2023-02-01

**Authors:** Jingyu Zhou, Zhiwen Ye, Peng Wei, Feng Yi, Min Ouyang, Shilang Xiong, Yayun Liu, Jintang Li, Min Liu, Hanrui Xi, Qianyi Peng, Long Xiong

**Affiliations:** ^1^Department of Orthopaedics, Second Affiliated Hospital of Nanchang University, Nanchang, Jiangxi, China; ^2^Department of Critical Care Medicine, Xiangya Hospital, Central South University, Changsha, China; ^3^Hunan Provincial Clinical Research Center for Critical Care Medicine, Xiangya Hospital, Central South University, Changsha, China; ^4^National Clinical Research Center for Geriatric Disorders (Xiangya Hospital), Xiangya Hospital, Central South University, Changsha, China; ^5^Department of Orthopaedics, The First Affiliated Hospital of Nanchang University, Nanchang, Jiangxi, China; ^6^Department of Orthopaedics, People's Hospital Affiliated with Nanchang University, Nanchang, Jiangxi, China

**Keywords:** basal metabolic rate, osteoporosis, genome-wide association study, Mendelian randomization, aging

## Abstract

**Purpose:**

Basal metabolic rate may play a key role in the pathogenesis and progression of osteoporosis. We performed Mendelian random analysis to evaluate the causal relationship between basal metabolic rate and osteoporosis.

**Methods:**

Instrumental variables for the basal metabolic rate were selected. We used the inverse variance weighting approach as the main Mendelian random analysis method to estimate causal effects based on the summary-level data for osteoporosis from genome-wide association studies.

**Results:**

A potential causal association was observed between basal metabolic rate and risks of osteoporosis (odds ratio = 0.9923, 95% confidence interval: 0.9898–0.9949; *P* = 4.005^e − 09^). The secondary MR also revealed that BMR was causally associated with osteoporosis (odds ratio = 0.9939, 95% confidence interval: 0.9911–0.9966; *P* = 1.038^e − 05^). The accuracy and robustness of the findings were confirmed using sensitivity tests.

**Conclusion:**

Basal metabolic rate may play a causal role in the development of osteoporosis, although the underlying mechanisms require further investigation.

## 1. Introduction

Osteoporosis (OP) is a systemic disease, which is based on low bone mass and microstructural destruction of bone tissue. The disease has become increasingly common worldwide with the growth of the elderly population (1). According to the European Demographic Report, the population aged 65 years and older accounts for approximately 20% of the total population, and it is estimated that by 2050, this proportion will reach 30% (2). In 2015, there were an estimated 20 million patients with OP in the European Union and Sweden (EU6). Among them, the prevalence rates of OP among men and women aged 50 or above is 6.8 and 22.5%, respectively (3). OP can lead to many complications such as chronic pain, decreased activities of daily living, and fractures, which can greatly reduce the quality of life of patients and increase mortality and medical care costs (3, 4). Fracture-related costs for the EU6 in 2017 were estimated to total €37.5 billion (an increase of 27% from 2010), and are projected to increase to €47.4 billion in 2030 (3).

Many risk factors are associated with osteoporotic fractures, including low peak bone mass, hormonal disturbances, certain medications (e.g., glucocorticoids), smoking, low physical activity levels, low calcium and vitamin D intake, ethnicity, smaller body size, and personal or family history of fractures (5). Aging is associated with an increased rate of bone remodeling in both cancellous and cortical bone, combined with a negative remodeling balance, resulting in bone loss and disruption of bone microarchitecture (6, 7). This plays a critical role in the progression of OP. The most striking feature of aging is the reduction in metabolism and basal metabolic rate (BMR) (8). OP is considered an inevitable consequence of aging (9) and a retrospective study suggested that a decrease in BMR in women with type 2 diabetes mellitus was associated with postmenopausal OP (10). Similar to this case, a previous study revealed that BMR was strongly associated with bone density in elderly individuals (11). However, these conclusions were limited to postmenopausal women and their studies could not further to invest the causal relationship between BMR and OP. BMR can reflect the body's overall metabolism, which is very important for maintaining normal physiological function (12). During the processing of aging, the decline in function is inescapable. This process also could be happened to the bone. We notice that BMR decline and high incidence of OP in elderly is strikingly consistent. BMR may be a modifiable factor in reducing the incidence of osteoporosis.

Thus, to further identify the effect and causality between basal metabolic rate and OP, we conducted Mendelian randomization (MR) (13) to explain the relationship from a genetic perspective. MR is a new method for assessing the causal relationship between risk factors and outcomes, based on genetic variables. Unlike traditional statistical methods, it can significantly decrease potential confounding factors and provide a complex interpretation of results (14, 15).

## 2. Materials and methods

### 2.1. Study design

**Figure 1:**
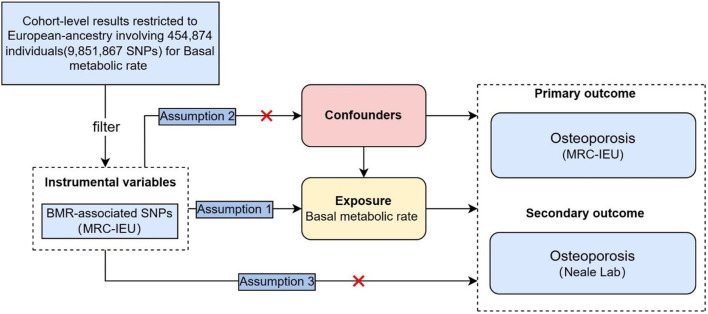
Schematic representation of the study.

Assumption 1 is that there is a strong correlation between the instrumental variable and the exposure factor; Assumption 2 is that the instrumental variable was not associated with any exposure-outcome confounding factor, and Assumption 3 is that the instrumental variable does not affect the outcome unless it is possible to do so through association with exposure. In this study, BMR was used as the exposure factor, single nucleotide polymorphisms (SNPs) significantly related to BMR were used as instrumental variables (IVs), and OP was the outcome variable. Here, we conducted a bidirectional two-sample MR analysis to estimate the causal effects in both directions of BMR and OP. A stratified analysis was performed to examine the causal effect of BMR on OP risk (Figure 1).

### 2.2. Genetic associations with outcomes

BMR and genome-wide association study (GWAS) data of OP were obtained from GWAS databases (http://gwas-api.mrcieu.ac.uk/, accessed on September 6, 2022). Therefore, additional ethical approval was not required. The OP data were divided into primary and secondary outcome data. The outcome data were obtained from the main result data of the public GWAS dataset (GWAS ID: ukb-b-12141). The dataset was constructed by the MRC Integrated Epidemiology Unit consortium using Biobank UK and included 462,933 Europeans (7,547 cases and 455,386 controls) with 9,851,867 SNPs. The secondary outcome data were obtained from a publicly available GWAS dataset (GWAS ID: ukb-a-87). This dataset was derived from the Neale Lab Consortium's summary data from the UK Biobank (UKB) and consisted of 337,159 Europeans (5,266 cases and 331,893 controls) with 10,894,596 SNPs. The details of the included GWAS are shown in Table 1.

**Table 1:** Summary of the GWAS included in this two-sample MR study.

**Exposure/** **outcomes**	**Dataset**	**Sample size**	**Number of SNPs**	**Population**	**Consortium**	**Sex**	**Year**
BMR	ukb-b-16446	454,874	9,851,867	European	MRC-IEU	Males and females	2018
OP	ukb-b-12141	462,933	9,851,867	European	MRC-IEU	Males and females	2018
OP	ukb-a-87	337,159	10,894,596	European	Neale Lab	Males and females	2017

### 2.3. Selection of instrumental variables

All selected SNPs were calculated as r^2^ values to evaluate linkage disequilibrium (LD). We first applied 546 SNPs associated with BMR at genome-wide significance (*P* < 5e^−8^). For any pair of SNPs with r^2^ > 0.001 (LD) in the range of 10,000 kb and with a minor allele frequency (MAF) > 5%, we retained those with the strongest associations on the exposure and obtained 521 independent SNPs. The potential confounders associated with the selected SNPs were analyzed using the PhenoScanner database (http://www.phenoscanner.medschl.cam.ac.uk/ accessed on September 14, 2022), and SNPs that were significantly associated with any known risk factors (age, inflammation, smoking, diabetes, renal function, vitamin D deficiency, and cancer, for example) for OP were removed. A total of 46 SNPs were excluded (Supplementary Table 1). Finally, the remaining SNPs and the related GWAS database measured the intersection point and obtained a significant correlation with the OP of the effective SNPs as instrumental variables. We conducted secondary analyzes on the data because the UKB individuals participated in both BMR and OP GWASs, which could cause weak instrumental bias due to sample overlap. We also examined possible pleiotropy of the selected SNPs using the outlier (MR-PRESSO) test, and abnormal SNPs were eliminated (16). Finally, the remaining SNPs were used for MR analysis.

We obtained the value of R^2^ from the MR Steiger directionality test. where N is the number of samples from the exposure GWAS, K is the number of SNPs in IV (when calculating a single SNP, the K value is 1), and R^2^ is the percentage of iron status variability explained by each SNP. R^2^ was calculated using Equation 1:


$$\begin{array}{l} R^{2}=2\times EAF\times \left(1-EAF\right)\times \beta^{2} \end{array}$$

In addition, we used the F-statistic to evaluate whether there was a weak IV bias (F-statistic < 10 was considered a weak IV) (17). The F-statistic was calculated using Equation 2:


$$\begin{array}{l} F=\frac{N-K-1}{K}\times \frac{R^{2}}{1-R^{2}} \end{array}$$

### 2.4. Statistical analysis

To determine whether there is a causal relationship between the genetic risk of exposure and outcome, we used inverse-variance weighting (IVW), MR-Egger, weighted median (WM), and weighted mode techniques to account for the likelihood of pleiotropy bias. The IVW method uses a meta-analysis approach to combine Wald estimates of each SNP to obtain an overall estimate of the effect of BMR on OP (18). Meanwhile, the fixed effects model or random effects model was selected according to heterogeneity (19). If significant heterogeneity (*P* < 0.05) was observed, a random-effects IVW model was applied. The MR-Egger method adds an intercept term to the IVW to express the average pleiotropy of instrumental variables, but the effective estimation of the EG method may be strongly influenced by external genetic variation, so the effective estimation is not accurate enough. The weighted-median method was supplemented to provide a robust and consistent estimate of the effect, even though nearly 50% of genetic variants were invalid instruments (20). The weighted mode method can only evaluate the causal validity according to the cluster with the largest number of SNPs, but cannot estimate the bandwidth parameter. After Bonferroni correction, the corrected *P* value was 0.0125 (*P* = 0.05/*N, N* = testing method number), which was statistically significant.

### 2.5. Sensitivity analysis

We used a sensitivity analysis to test and correct the robustness of the MR estimation. First, MR-Egger interceptions were used to evaluate the multiplicity of the SNPs. A lower multiplicity was considered when the intercept was closer to zero. Cochran's Q test was used to assess heterogeneity. Finally, to ensure the robustness of the results, we used the leave-one-out test to judge the stability of the MR results by excluding the IVs one by one.

## 3. Results

### 3.1. Genetic variables for BMR

In the primary group, IV (rs4974072 Rssobs = 1.176^e − 06^, *P* = 0.044) was identified as an outlier in the MR-PRESSO test and was excluded from subsequent analyzes. In the secondary group, no IVs were excluded using the MR-PRESSO test. Finally, we applied 439 valid SNPs as IVs in the secondary group and obtained 462 SNPs in the secondary group (Supplementary Tables 2, 3). Individual SNPs of iron status had F-statistics of more than 10, indicating that bias from weak instruments was unlikely. The details of IV are listed in Table 2.

**Table 2:** Sensitivity analysis of primary and secondary MR analyses.

**Outcome**	**Number**	**R^2^**	**F**	**Heterogeneity test**		**MR-Egger pleiotropy test**		**MR-PRESSO global**		**MR-PRESSO**
	**of IVs**							**pleiotropy test**		**Distortion Test**
				**Q**	***p*** **-value**	**Intercept**	***p*** **-value**	**RSSobs**	***p*** **-value**	***p*** **-Value**
Primary outcome	439	2.7527%	27.7101	598.4290	4.9487; e-07	−7.8420; e-05	0.0929	618.5921	< 5e-05	0.87425
Secondary outcome	462	2.8955%	29.3289	574.6058	2.3845; e-3	−5.7447; e-05	0.2339	577.3748	7e-04	*NA*

### 3.2. Primary MR analysis of BMR and OP

R^2^ was 2.7527%, and the F-statistic of a single SNP ranged from 11.56 to 256.80, indicating that causal association was less likely to be affected by weak instrumental variable bias. Cochran's Q test identified heterogeneity across the included IVs (Q = 598.4290, *P* = 4.9487^e − 07^), and a multiplicative random-effects model was used in this MR analysis. A significant causal association was observed in the IVW analysis between BMR and OP (MRC-IEU) (odds ratio [OR] = 0.9923, 95% confidence interval [95% CI]: 0.9898–0.9949; *P* = 4.005^e − 09^), and was replicated using WME (OR = 0.9926, 95% CI: 0.9888–0.9964; *P* = 1.176^e − 04^), MR Egger (OR = 0.9980, 95% CI: 0.9909–1.0051; *P* = 5.740^e − 01^), and weighted mode (OR = 1.0020, 95% CI: 0.9904–1.0136; *P* = 7.393^e − 01^). The robustness of the results was confirmed using a leave-one-out sensitivity test. No directional pleiotropy was found in the MR-Egger regression (intercept = −7.8420^e − 05^, se = 4.6563^e−^05, *P* = 0.0929).

### 3.3. Secondary MR analysis of BMR and OP

R^2^ was 2.8955%, and the F-statistic of a single SNP ranged from 11.56 to 257.10, indicating that causal association was less likely to be affected by weak instrumental variable bias. Cochran's Q test identified heterogeneity across the included IVs (Q = 574.6058, *P* = 2.3845^e − 4^), and a multiplicative random-effects model was used in this MR analysis. The IVW analysis reported a significant causal relationship between BMR and OP (OR = 0.9939, 95% CI: 0.9911–0.9966; *P* = 1.038^e − 05^), which was consistent with the findings of the WME (OR = 0.9932, 95% CI: 0.9893–0.9972; *P* = 7.765^e − 04^), MR-Egger analysis (OR = 0.9979, 95% CI: 0.9907–1.0051; *P* = 5.620^e − 01^), and weighted mode (OR = 0.9946, 95% CI: 0.9843–1.0049; *P* = 3.046^e − 01^). The robustness of the results was confirmed using a leave-one-out sensitivity test. No directional pleiotropy was found in the MR-Egger regression (intercept = −5.7447^e − 05^, se = 4.8194^e − 05^, *P* = 0.2339).

**Figure 2:**
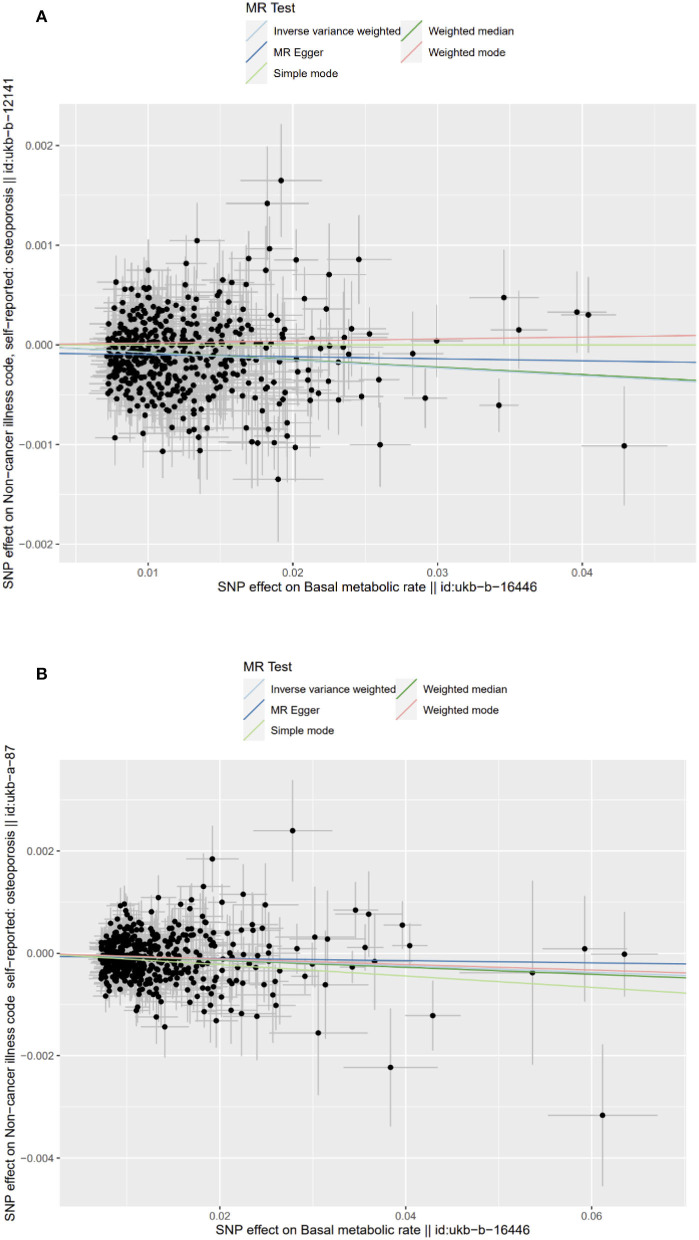
Scatter plots of causality. The slope of each line corresponding to the estimated MR effect in different models. **(A)** Primary outcome (ukb-b-12141); **(B)** Secondary outcome (ukb-a-87).

**Figure 3:**
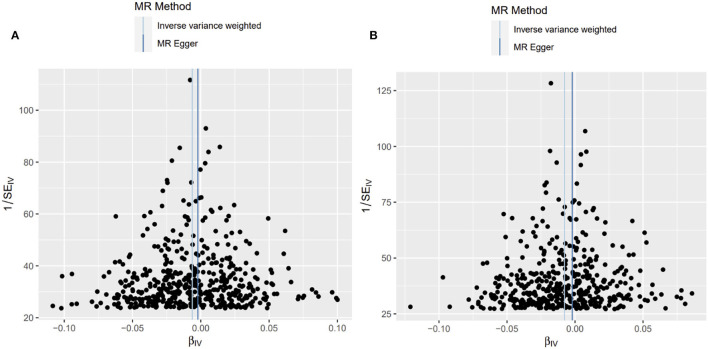
Funnel plot of causality. **(A)** Primary outcome (ukb-b-12141); **(B)** Secondary outcome (ukb-a-87).

**Figure 4:**
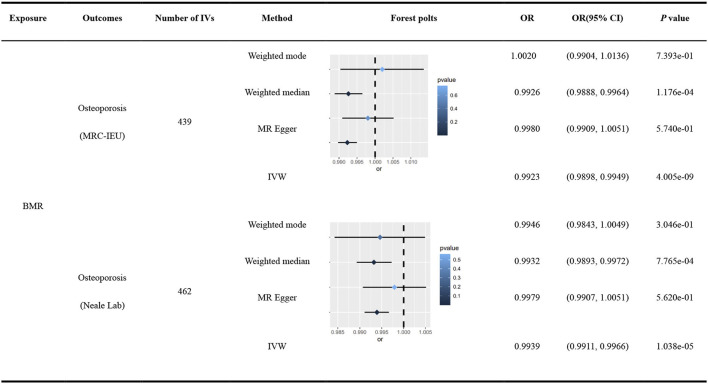
Causal estimates given as odds ratios (ORs) and 95% confidence intervals for the effect of basal metabolic rate on osteoporosis by different consortium.

The scatter and forest plots of the causal relationships between BMR and the risk of OP are shown in Figure 2 and Supplementary Figures 1, 2. The funnel plot (Figure 3) shows that when a single SNP is used as an IV, the points representing causal effects are symmetrically distributed, which indicates that causal effects are less likely to be affected by potential bias. In the leave-one-out analysis (Supplementary Figures 3, 4), the removal of each SNP in turn has little effect on the results, indicating that there is no single SNP which has a significant influence on the overall causal effect estimation. The details of the sensitivity analysis are presented in Table 2. Detailed causal effect estimates for the associations between exposure and outcomes in different models are illustrated in Figure 4.

## 4. Discussion

Few studies have reported a relationship between BMR and OP. In our study, we used two-sample MR analysis to assess the causal association between BMR and OP. We found a significant causal association between the genetic risk of BMR and OP, showing an average of 0.8% decreased risk of OP per 1-SD increment in BMR and this conclusion was also applicable to the secondary group (OR = 0.9939, 95% CI: 0.9911–0.9966).

Osteoporosis is an enormous and growing public health problem. As a common disease in the elderly, especially postmenopausal women (21) OP is considered to be an important factor for fractures and strategies to prevent OP could decrease half of all hip fractures (22). Interestingly, an observational study of osteoporosis in postmenopausal women showed that BMR was related to BMD independent of age and patients with OP had a significantly lower BMR compared to those non-OP. Similarly, in the process of human aging, there is an inevitable decline in BMR, and this alteration is strikingly similar to the high incidence of osteoporosis in the elderly. We calculate that there is a causal relationship between BMR and OP and that the increase in BMR can reduce the incidence of osteoporosis. Thus, we conducted a two-sample Mendelian randomized study, and our study provided evidence supporting a potential causal relationship between low BMR levels and an increased risk of osteoporosis. MR-Egger's causal estimate and weighted median analysis are consistent with IVW analysis, indicating that the causal effect is reliable. Our study confirmed that an increased BMR could promote the reduction of OP from a genetic perspective and provide a positive causality of BMR in reducing OP and this conclusion is not limited to postmenopausal women. This provides a new insight for preventing and treating OP at a reversible stage, which may significantly reduce the risk of fracture and related burdens in patients.

Over the past decades, research on BMR has mainly focused on obesity and metabolic-related diseases (23), and it has been well applied in the treatment of diabetes. BMR plays a key role in maintaining normal structure and function, and an imbalance in metabolic demands and BMR may be closely related to clinical diseases. The decrease in physical activity with the onset of the aging process masks the lifestyle of the entire elderly population, resulting in a substantial increase in sedentary time (24, 25). Maybe it is an important reason for the decline in BMR in the elderly and leads to an increased OP. Guidelines (26, 27) endorse lifelong physical activity (both weight-bearing exercise and muscle-strengthening exercise) for osteoporosis prevention. This may be achieved by improving BMR. Although frequency and intensity of activity are not specified in guidelines, a common clinical practice is to recommend both 30 min of walking per day and gentle resistance exercises (4). And we also noticed that counter-resistance exercises are effective in maintaining bone mineral mass and bone mineral density since mechanical stimulation promotes the formation of new bone tissue (28). But high-intensity physical activity is limited to the elderly.

Once humans enter the aging stage, subsequently a decrease in total energy consumption and energy availability follows (29, 30). More importantly, energy metabolism is highly correlated with body composition. Once energy metabolism changes, functional changes will inevitably be affected. According to our results, we speculate that the decrease of BMR may affect bone metabolism and lead to the occurrence of osteoporosis, but how to realize this process needs more basic and clinical research to further clarify.

Drugs used to treat OP, such as antiresorptive drugs, inhibit the recruitment and activity of osteoclasts, thereby rebalancing bone resorption and synthesis (31). In patients with OP, the normal balance is compromised, leading to an abundance of missing reconstructed osteons and a decrease in bone quality. At present, the recommended first-line drugs for treating osteoporosis include bisphosphonate and teriparatide, which have been confirmed effective in clinical practice, but there are the following concerns. Teriparatide can improve osteoporosis by increasing bone density, but it needs to be taken for a long time. Once stopped, the curative effect drops rapidly and even accelerates bone loss. Bisphosphate is one of the most common drugs for OP in clinical practice. Bisphosphonate treatment is associated with 40–70% reductions in vertebral fractures and 40–50% reductions in hip fractures. However, it is limited in patients with severe renal damage. bisphosphonate may also lead to gastrointestinal irritation and musculoskeletal abnormalities. Supplementation of bone anabolic elements (such as calcium and vitamin D), may help to ameliorate this, but the benefit of supplementation is only observed in individuals with low intake and impaired absorption (7). These routine measures can slow OP in the elderly, but they do not restore BMD and are not adequate treatments for patients with an elevated risk of fracture. Our study further confirmed the causal relationship between BMR and OP in terms of genetic risk, enhancing the importance of BMR in bone development, bone metabolism, and related bone diseases and provides new targets for the treatment and research of OP in the future.

## 5. Conclusion

In conclusion, our results suggest that higher BMR may reduce the risk of OP. Interventions with modifiable goals to improve BMR may help reduce the burden of this chronic disease.

## 6. Limitation

This study has several limitations. First, the OP cases in this study were from self-reported osteoporosis patients in the UKB. Self-reported disease status may miss cases of OP and mistakenly include other bone diseases. Second, our results apply only to European lineages, and the effect of BMR on OP in other lineages has not yet been studied. Second, it is the only publicly available GWAS on OP. We did not report on the subtypes of OP. These include postmenopausal OP, senile OP, and drug-induced OP. Third, it is impossible to further classify OP and conduct a stratified MR analysis based on the subtypes of OP. This would be helpful in drawing more accurate causal inferences, with more control over potential confounders. In addition, since limited to the database, we failed to assess the health status of the sample population, which may lead to potential confounding bias. Finally, in this study, BMR genes were selected as instrumental variables to calculate the causal relationship between BMR and OP. No study has explored the mechanism underlying the relationship between BMR and OP. Assessing the role of BMR in OP from a genetic perspective may only partially explain the role of BMR in OP.

## Data availability statement

The original contributions presented in the study are included in the article/Supplementary material, further inquiries can be directed to the corresponding authors.

## Ethics statement

Ethical review and approval was not required for the study on human participants in accordance with the local legislation and institutional requirements. The patients/participants provided their written informed consent to participate in this study. Written informed consent was not obtained from the individual(s), nor the minor(s)' legal guardian/next of kin, for the publication of any potentially identifiable images or data included in this article.

## Author contributions

JZ and ZY finished the original manuscript. LX and QP designed the study and supervised the study. All authors contributed to the article and approved the submitted version.

## References

[1] Consensus development conference: diagnosis and treatment of osteoporosis. Am J Med. (1993) 94:646–50. 10.1016/0002-9343(93)90218-E 8506892

[2] Tornero-Quiñones I Sáez-Padilla J Espina Díaz A Abad Robles MT Sierra Robles Á. Functional ability, frailty and risk of falls in the elderly: relations with autonomy in daily living. Int J Environ Res Public Health. (2020) 17:1006. 10.3390/ijerph17031006 32033397 PMC7037456

[3] Borgström F Karlsson L Ortsäter G Norton N Halbout P Cooper C. Fragility fractures in Europe: burden, management and opportunities. Arch Osteoporos. (2020) 15:59. 10.1007/s11657-020-0706-y 32306163 PMC7166207

[4] Ensrud KE Crandall CJ. Osteoporosis. Ann Intern Med. (2017) 167:ITC17–ITC32. 10.7326/AITC201708010 28761958

[5] Lane NE. Epidemiology, etiology, and diagnosis of osteoporosis. Am J Obstet Gynecol. (2006) 194:S3–11. 10.1016/j.ajog.2005.08.047 16448873

[6] Compston JE Mellish RW Garrahan NJ. Age-related changes in iliac crest trabecular microanatomic bone structure in man. Bone. (1987) 8:289–92. 10.1016/8756-3282(87)90004-4 3426887

[7] Compston JE McClung MR Leslie WD. Osteoporosis. Lancet. (2019) 393:364–76. 10.1016/S0140-6736(18)32112-3 30696576

[8] Denckla WD. Role of the pituitary and thyroid glands in the decline of minimal O_2_ consumption with age. J Clin Invest. (1974) 53:572–81. 10.1172/JCI107592 11344572 PMC301501

[9] Khosla S Hofbauer LC. Osteoporosis treatment: recent developments and ongoing challenges. Lancet Diabetes Endocrinol. (2017) 5:898–907. 10.1016/S2213-8587(17)30188-2 28689769 PMC5798872

[10] Gundmi S Maiya AG Bhat AK Hande MH Kumar AS. Screening for basal metabolic rate and visceral fat among postmenopausal osteoporosis with type 2 diabetes mellitus. Diabetes Metab Syndr. (2019) 13:981–4. 10.1016/j.dsx.2019.01.003 31336555

[11] Choi JW Pai SH. Bone mineral density correlates strongly with basal metabolic rate in postmenopausal women. Clin Chim Acta. (2003) 333:79–84. 10.1016/S0009-8981(03)00190-6 12809738

[12] Macgregor AG Wayne EJ Miller H Goodwin JFBMR. And radioiodine tests in diagnosis of thyroid disease. Lancet. (1952) 1:616–7. 10.1016/S0140-6736(52)90142-6 14909499

[13] Emdin CA Khera AV Kathiresan S. Mendelian randomization. JAMA. (2017) 318:1925–6. 10.1001/jama.2017.17219 29164242

[14] Zheng J Baird D Borges M-C Bowden J Hemani G Haycock P. Recent developments in mendelian randomization studies. Curr Epidemiol Rep. (2017) 4:330–45. 10.1007/s40471-017-0128-6 29226067 PMC5711966

[15] Lee K Lim C-Y. Mendelian randomization analysis in observational epidemiology. J Lipid Atheroscler. (2019) 8:67–77. 10.12997/jla.2019.8.2.67 32821701 PMC7379124

[16] Verbanck M Chen C-Y Neale B Do R. Detection of widespread horizontal pleiotropy in causal relationships inferred from mendelian randomization between complex traits and diseases. Nat Genet. (2018) 50:693–8. 10.1038/s41588-018-0099-7 29686387 PMC6083837

[17] Staiger DO Stock JH. Instrumental variables regression with weak instruments. Economet eJournal. (1994). 10.3386/t0151

[18] Bowden J Del Greco M F Minelli C Davey Smith G Sheehan N Thompson J. Framework for the investigation of pleiotropy in two-sample summary data mendelian randomization. Stat Med. (2017) 36:1783–802. 10.1002/sim.7221 28114746 PMC5434863

[19] Zheng J Frysz M Kemp JP Evans DM Davey Smith G Tobias JH. Use of mendelian randomization to examine causal inference in osteoporosis. Front Endocrinol (Lausanne). (2019) 10:807. 10.3389/fendo.2019.00807 31824424 PMC6882110

[20] Bowden J Davey Smith G Haycock PC Burgess S. Consistent estimation in mendelian randomization with some invalid instruments using a weighted median estimator. Genet Epidemiol. (2016) 40:304–14. 10.1002/gepi.21965 27061298 PMC4849733

[21] Management of Osteoporosis in Postmenopausal Women: The 2021 Position Statement of The North American Menopause Society” Editorial Panel. Management of osteoporosis in postmenopausal women: the 2021 position statement of the north american menopause society. Menopause. (2021) 28:973–97. 10.1097/GME.0000000000001831 34448749

[22] Odén A McCloskey EV Johansson H Kanis JA. Assessing the impact of osteoporosis on the burden of hip fractures. Calcif Tissue Int. (2013) 92:42–9. 10.1007/s00223-012-9666-6 23135744

[23] Guan L Li T Wang X Yu K Xiao R Xi Y. Predictive roles of basal metabolic rate and body water distribution in sarcopenia and sarcopenic obesity: the link to carbohydrates. Nutrients. (2022) 14:3911. 10.3390/nu14193911 36235562 PMC9571591

[24] Paintin J Cooper C Dennison E. Osteosarcopenia. Br J Hosp Med (Lond). (2018) 79:253–8. 10.12968/hmed.2018.79.5.253 29727228 PMC5963675

[25] Harvey JA Chastin SFM Skelton DA. How sedentary are older people? A systematic review of the amount of sedentary behavior. J Aging Phys Act. (2015) 23:471–87. 10.1123/japa.2014-0164 25387160

[26] Cosman F de Beur SJ LeBoff MS Lewiecki EM Tanner B Randall S. Clinician's guide to prevention and treatment of osteoporosis. Osteoporos Int. (2014) 25:2359–81. 10.1007/s00198-014-2794-2 25182228 PMC4176573

[27] Camacho PM Petak SM Binkley N Clarke BL Harris ST Hurley DL. American association of clinical endocrinologists and american college of endocrinology clinical practice guidelines for the diagnosis and treatment of postmenopausal osteoporosis−2016–executive summary. Endocr Pract. (2016) 22:1111–8. 10.4158/EP161435.GL 27643923

[28] Troy KL Mancuso ME Butler TA Johnson JE. Exercise early and often: effects of physical activity and exercise on women's bone health. Int J Environ Res Public Health. (2018) 15:878. 10.3390/ijerph15050878 29710770 PMC5981917

[29] Soysal P Ates Bulut E Yavuz I Isik AT. Decreased basal metabolic rate can be an objective marker for sarcopenia and frailty in older males. J Am Med Dir Assoc. (2019) 20:58–63. 10.1016/j.jamda.2018.07.001 30122323

[30] Abizanda P Romero L Sánchez-Jurado PM Ruano TF Ríos SS Sánchez MF. Energetics of aging and frailty: the fradea study. J Gerontol A Biol Sci Med Sci. (2016) 71:787–96. 10.1093/gerona/glv182 26463762 PMC4888388

[31] Reid IR Billington EO. Drug therapy for osteoporosis in older adults. Lancet. (2022) 399:1080–92. 10.1016/S0140-6736(21)02646-5 35279261

