# Supplementary figures and images for: Effect of basal metabolic rate on osteoporosis: A Mendelian randomization study
Source: Front Public Health. 2023 Feb 1;11:1096519. doi: 10.3389/fpubh.2023.1096519 (PMC9929187; doi:10.3389/fpubh.2023.1096519)

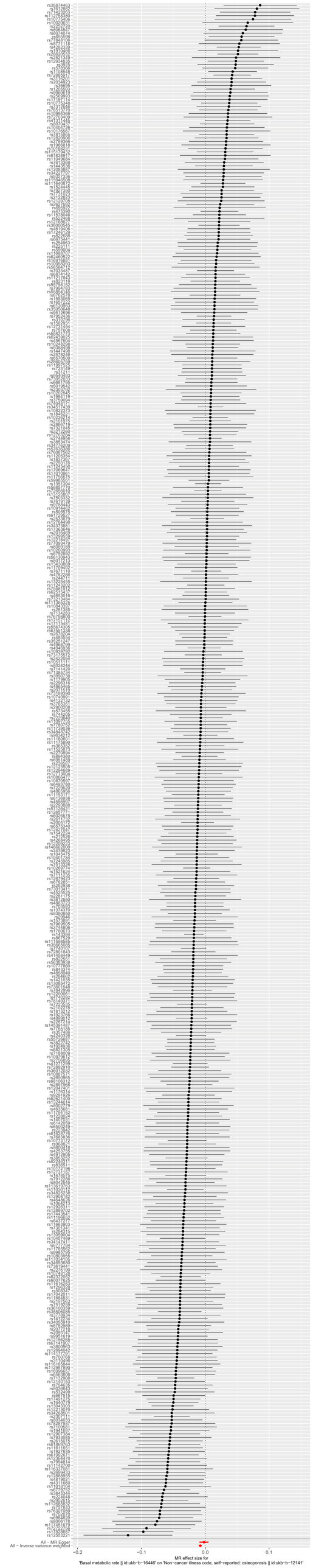

Supplement: Supplementary file 1 [file Image_1.TIFF]

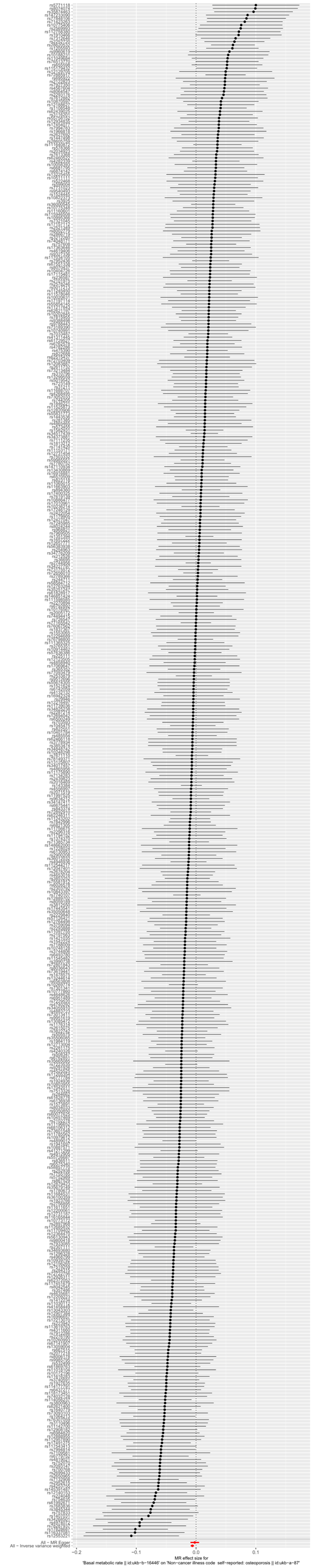

Supplement: Supplementary file 2 [file Image_2.TIFF]

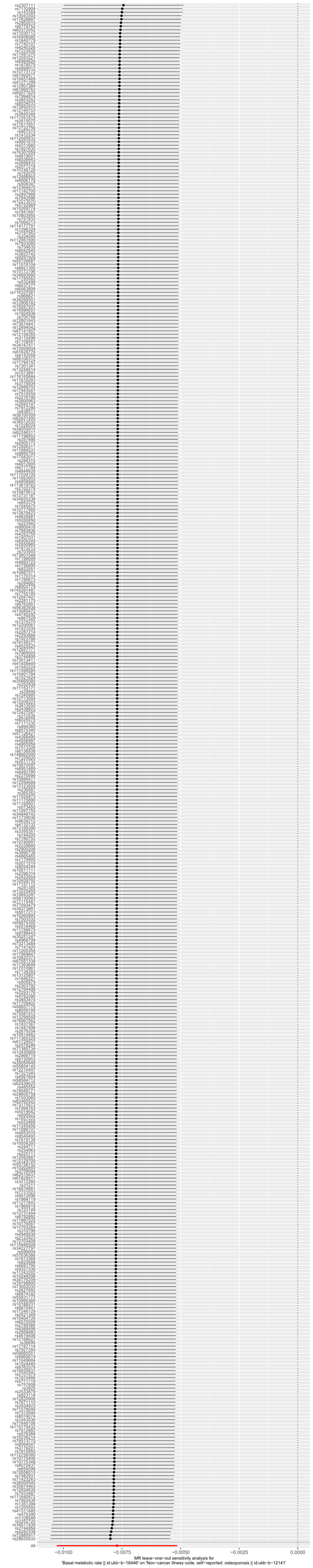

Supplement: Supplementary file 3 [file Image_3.TIFF]

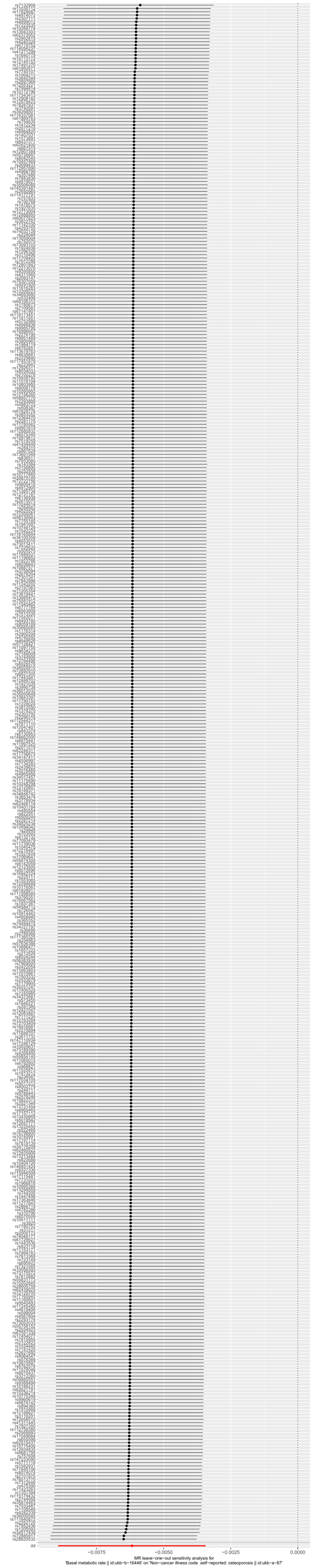

Supplement: Supplementary file 4 [file Image_4.TIFF]
